# The Effect of Blood Transfusion on the Survival of Children with Both Severe Anemia and Bacterial Meningitis

**DOI:** 10.4269/ajtmh.22-0650

**Published:** 2023-02-27

**Authors:** Tuula Pelkonen, Irmeli Roine, Manuel Cruzeiro, Kirsi Jahnukainen, Heikki Peltola

**Affiliations:** ^1^ Pediatrics, University of Helsinki and Helsinki University Hospital, Helsinki, Finland;; ^2^ New Children’s Hospital, Pediatric Research Center, Helsinki, Finland;; ^3^ Hospital Pediátrico David Bernardino, Luanda, Angola;; ^4^ Faculty of Medicine, University Diego Portales, Santiago, Chile

## Abstract

In areas with suboptimal resources, blood transfusion may not be feasible even when mandatory for severely anemic children with a life-threatening disease. We evaluated how much not having received a transfusion affected the survival in 171 children with an admission blood hemoglobin level of < 6 g/dL and bacterial meningitis in Luanda, Angola. Of these children, 75% (128 of 171) had received a blood transfusion during hospitalization, but 25% (43 of 171) had not. Within the first week, 33% of patients (40 of 121) with transfusion and 50% (25 of 50) without a transfusion died (*P* = 0.04). Early transfusion (days 1–2 of hospitalization) prolonged the time of survival from a median of 132 hours [interquartile range (IQR), 15–168] to 168 hours (IQR, 69–168; *P* = 0.004), and had odds of 0.49 (95% CI, 0.25–0.97; *P* = 0.040) for death compared with no transfusion. The effect of transfusion/no transfusion at any time during hospitalization on mortality within 30 days, and prolongation of the time of survival were similar to early transfusion but showed even clearer benefits. Our results emphasize the value of timely transfusion in facilities that care for severely anemic children with severe infections to maximize their chances of survival.

## INTRODUCTION

In sub-Saharan Africa, anemia is highly prevalent in children.^1^^,^^2^ Moderate to severe anemia is a known complication in severe disease and triples the odds for death in childhood bacterial meningitis (BM).^1^^,^^2^ The principal causes of anemia in sub-Saharan Africa, in order of importance, but often combined, include malaria, other parasitic infections, sickle cell disease, undernutrition, and inadequate iron intake.^3^

Opposite to the other risks for adverse outcomes from BM, severe anemia is mitigable or reversable by blood transfusion.^4^ Other fundamental risks, such as the degree of severity on admission, treatment delay, undernutrition, or pneumococcal etiology, cannot be reversed, or instantly bettered.^4^

Red cell transfusion improves the anemic patient’s oxygen carrying capacity to tissues that may be already hypoxic from processes inherent to severe infections such as edema and circulatory insufficiency.^5^ In children with severe anemia, blood transfusion has been shown to improve cerebral tissue oxygenation.^6^ Potential risks of transfusion include bacterial and viral contamination, acute or delayed hemolytic reactions, and circulatory overload.^7^^,^^8^ Alloimmunization and immunomodulatory effects may have long-term consequences.^7^

The WHO criterion for a blood transfusion is a hemoglobin level < 4 g/dL, but in the presence of emergency signs (severe respiratory distress, shock, coma, convulsions, and severe dehydration), the level is 4 to 6 g/dL.^9^ At our study site in Luanda, Angola, most clinicians follow the local < 5 g/dL recommendation; but for an especially ill child, the level of < 6 g/dL is used. In areas with suboptimal resources, realities such as availability of blood or functioning of the blood bank, especially at night, may delay a transfusion or sometimes even make it unfeasible.^7^

The benefit and harm of transfusion in children have been studied in uncomplicated severe anemia,^10^^,^^11^ in severe febrile illness, and in critical care in general,^1^ but not regarding the survival of severely anemic children with an acute life-threatening bacterial infection such as BM. This observation spurred us to take a second look at the data collected during our two large clinical trials on BM, now focusing on the patients with an admission hemoglobin level of < 6 g/dL.^12^^,^^13^ Here we describe how blood transfusion affected their survival.

## MATERIALS AND METHODS

This is a secondary analysis of data collected prospectively from two clinical trials aimed at improving BM prognosis in 2005 to 2008 (#ISRCTN62824827) and 2012 to 2017 (#NCT01540838). Both studies were approved before study start by the Ethics Committee of Hospital Pediátrico David Bernardino, Luanda, Angola (June 22, 2005 and January 19, 2012). The patients were enrolled after written or oral consent was obtained from a guardian. All patients received the same antimicrobial treatment (cefotaxime 250 mg/kg/day for a minimum of 7 days) and were monitored from enrollment until death or discharge.

The inclusion criteria for our study were to have a confirmed BM diagnosis and a known blood hemoglobin level on admission.

Details of the clinical trials (#ISRCTN62824827 and #NCT01540838) have been published previously.^12^^,^^13^ In brief, patients with symptoms and signs suggestive of BM aged 2 months to 15 years were enrolled consecutively if consent was provided by a legal guardian. The children were randomized to receive treatment alternatives (cefotaxime stat or by infusion, and with or without acetaminophen), which ultimately did not improve outcomes significantly.

The data collected prospectively by the study personal included patient characteristics, admission findings, course of disease, and outcome according to predefined criteria. All deaths were registered according to the time (in hours) elapsed from the institution of antimicrobial treatment. For blood transfusion, the data indicated the day of hospitalization when administered; the day of admission was considered day 1. We did not have a record of the time in hours between blood transfusion and deaths.

In the Pediatric Hospital of Luanda, the blood used is provided by the National Blood Transfusion Service. Blood is obtained from voluntary, anonymous donors or family donors.

A blood transfusion consisted of 15 mL/kg of packed red blood cells given over 4 hours. The blood was tested for HIV, hepatitis B, and syphilis. Furosemide was given only to children with signs of fluid overload.

### Formation of compared groups.

Blood hemoglobin level was measured on days 1 to 2 of hospitalization, and most transfusions took place on the same days, but sometimes later, and some patients had more than one transfusion. Within these premises we searched for a clinically relevant question to test how transfusion affected survival. Motivated by previous studies, we decided to compare the patients with and without an early transfusion on days 1 to 2 (= early transfusion) versus survival within days 1 to 7.^14^ Secondarily, we compared the patients with or without a transfusion at any time during hospitalization versus death within 30 days since admission.

### Statistics.

The descriptive data are expressed in counts and percentages, or medians with interquartile range (IQR), whichever appropriate. The potential differences in the basic characteristics between groups with or without early transfusion were examined by Mann Whitney *U* tests or χ^2^ tests in accordance with the type of data.

The associations between transfusion and survival were examined in two ways. The first was by a Kaplan-Meier survival analysis along with log-rank tests comparing the time of survival in hours in the groups with or without transfusion. In these analyses, patients alive on day 7 (= minimum days of hospitalization) were assigned a time of survival of 168 hours (24 hours × 7 days). The second way was by calculating the odds ratio (OR) with 95% CIs for death within the first week in the hospital according to the receipt or no receipt of an early transfusion. The Kaplan-Meier analysis was performed first without stratification, and then was stratified by admission severity as categorized by a Glasgow Coma Scale score < 13 points (= severe), compared with ≥ 13 points (= less severe). The OR calculations were adjusted by admission severity.

The same analytic approach was applied to the secondary question regarding the receipt or no receipt of a transfusion at any time during hospitalization versus mortality within 30 days since admission. Patients discharged alive before 30 days were assigned a time of survival by multiplying days of hospitalization by 24 hours, whereas those who were alive in the hospital on day 30 were assigned a time of survival of 720 hour (30 days × 24 hours). A *P* value < 0.05 was considered significant. The calculations were performed using Statview version 5.1 (SAS Institute, Cary, NC) and the figures were prepared with R version 4.1.3 (R Foundation for Statistical Computing, Vienna, Austria) using the survminer-package.^15^

## RESULTS

Of the 822 patients with confirmed BM, 98% (804 of 822) had a known admission hemoglobin level and, of them, in 21% (171 of 804) it was < 6 g/dL. Of the patients with hemoglobin < 6 g/dL, 75% (128 of 171) had received blood during hospitalization. The transfusion was administered on median day 1 of hospitalization (IQR, 0.5; range, 1–7 days), and in 30% (38 of 128) it was repeated later. In 75% of the patients (96 of 128), the transfusion/first transfusion took place on day 1 of hospitalization; in 95% (121 of 128), on day 1 or 2 (= early transfusion); and in 5% (7 of 128), at a later date.

Of patients with an admission hemoglobin level < 6 g/dL, 29% (50 of 171) did not receive an early transfusion and 25% (43 of 171) had no transfusion during hospitalization. Why they did not was unregistered. Possible reasons could be that blood was not available soon enough or the hemoglobin result was delayed. Some clinicians may have adhered to the < 5 g/dL limit because the patients without a transfusion (Table 1) had a greater hemoglobin level compared with the those who did receive a transfusion (median, 5.1 g/dL versus 4.8 g/dL, respectively; *P* = 0.004).

**Table 1:** Differences in basic characteristics in patients with bacterial meningitis and admission hemoglobin < 6 g/dL according to early blood transfusion on days 1 to 2

Variable	Early transfusion		Without transfusion		*P* value
	*n*	Median (IQR)	*n*	Median (IQR)	
Age, months	121	12 (7–24)	50	7 (5–28)	0.09
Symptoms before admission, days	119	6 (4–8)	50	5 (3–10)	0.26
Weight-for-age *z*-score (SD)	121	−1.8 (−0.6 to −2.7)	50	−1.07 (−0.2 to −2.2)	0.03
Blood					
White cell count, ×10^9^/L	61	19.1 (10.6–28.7)	21	16.6 (8.9–24.8)	0.34
Hemoglobin days 1–2, g/dL	121	4.8 (4.2–5.4)	50	5.1 (4.8–5.5)	0.004
Glucose, mg/dL	120	86 (69–106)	50	80 (61–96)	0.07
C-reactive protein day 1, mg/L	97	161 (133–161)	31	161 (159–169)	0.07
Cerebrospinal fluid					
White cell count, ×10^9^/L	121	1.2 (0.3–3.3)	50	1.9 (0.2–3.8)	0.51
Glucose, mg/dL	116	10 (5–20)	50	15 (6–25)	0.23
Protein, mg/dL	29	197 (103–235)	19	220 (180–249)	0.20
Systolic blood pressure, mm Hg	118	98 (90–109)	44	96 (90–105)	0.81
Diastolic blood pressure, mm Hg	118	60 (50–70)	44	60 (49–66)	0.56
Heart rate, beats/minute	116	128 (119–144)	46	127 (112–144)	0.58
Respiratory rate, breaths/minute	102	44 (36–55)	39	40 (35–52)	0.46
Capillary filling time, seconds	38	2 (1–3)	23	2 (1–2)	0.87
Oxygen saturation, %	75	97 (94–98)	25	97 (95–98)	0.70
Etiology					
*Haemophilus influenzae*, *n* (%)		45 (40)		12 (26)	0.01
*Neisseria meningitidis*, *n* (%)		1 (1)		5 (11)	
Streptococcus pneumoniae, *n* (%)		51 (45)		21 (46)	
Other, *n* (%)		16 (14)		8 (17)	
Sickle cell disease, *n* (%)	44	22 (50)	16	5 (31)	0.20
GCS score, points (3–15, best)	113	12 (8–15)	47	10 (7–15)	0.13
GCS outcome score, points (1–5, best)	111	3 (1–5)	50	1 (1–5)	0.16

GCS = Glasgow Coma Scale; IQR = interquartile range; *n* = number of patients for whom the information was available; *n* = number of patients with the condition (percentage).

At presentation to hospital, the early-transfusion versus no-early-transfusion groups did not differ in their systolic or diastolic blood pressure, pulse frequency, or capillary filling times (Table 1). Axillary temperature at admission was recorded in 99% of the patients (169 of 171). We found no evidence of more deaths (the only specific harm that we noted) in febrile patients who received a transfusion versus afebrile ones.

### Survival and comparisons of the time of survival.

Of the 121 patients who received an early transfusion, 33% (40 of 121) died within 7 days, compared with every other child, 50% (25 of 50; *P* = 0.04) without an early transfusion. The median times of survival were 132 hours (IQR, 15–168) and 168 hours (IQR, 69–168) in patients without and with an early transfusion, respectively (*P* = 0.004).

**Figure 1.**
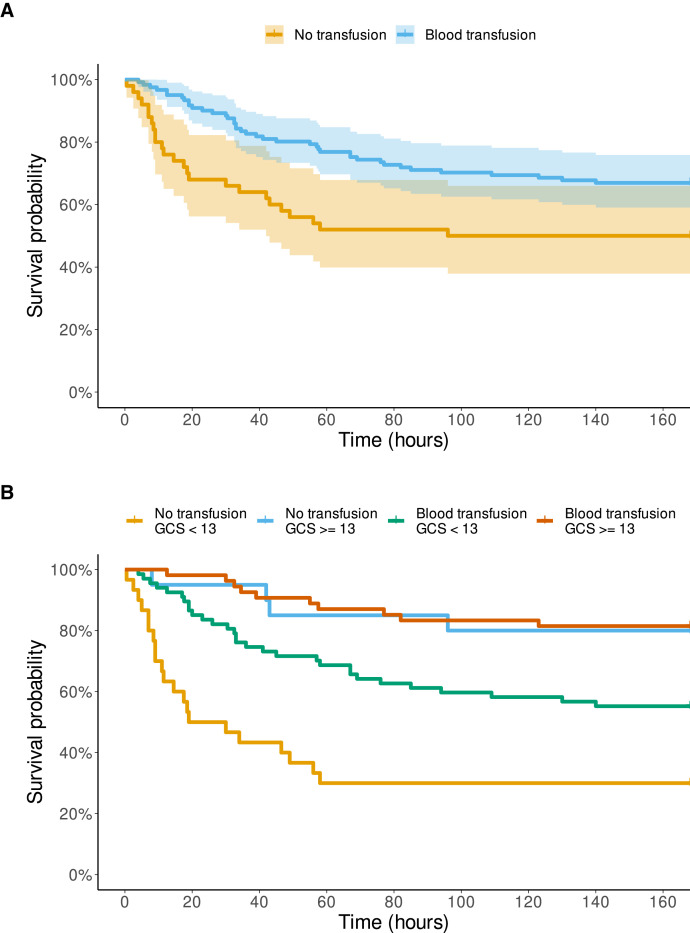
Time of survival in hours of the 171 patients with bacterial meningitis with admission hemoglobin < 6 g/dL with and without a blood transfusion on day 1 to 2 during the first 7 days (= 168 hours) of hospitalization. Glasgow Coma Scale (GCS) score on admission (3–15, best). (**A**) Without stratification. The colored area represents 95% confidence intervals. (**B**) Stratified by severity of disease.

As depicted in Figure 1A, the patients with an early transfusion survived longer than those without (log-rank Mantel-Cox, *P* = 0.008). An early transfusion reduced the odds (Table 2) for death within 7 days (OR, 0.49; 95% CI, 0.25–0.97; *P* = 0.04).

**Table 2:** The odds for death on days 1 to 7 and 1 to 30 of hospitalization according to the timing of a blood transfusion, either on day 1 or 2 or any time during hospitalization vs. not having received a transfusion for 171 children with bacterial meningitis and admission hemoglobin < 6 g/dL

Time of death	Early transfusion, days 1–2				Transfusion anytime during hospitalization			
	OR	95% CI	aOR	95% CI	OR	95% CI	aOR	95% CI
Days 1–7	0.49	0.25–0.97	0.59	0.29–1.20	0.33	0.16–0.67	0.38	0.18–0.81
Days 1–30	0.42	0.22–0.83	0.49	0.24–1.01	0.27	0.13–0.56	0.30	0.14–0.66

aOR = adjusted odds ratio (adjusted by admission severity); OR = odds ratio.

When the analysis was stratified by disease severity (Figure 1B), the patients with severe disease (Glasgow Coma Scale score < 13 points) and without a transfusion showed the shortest survival (log-rank Mantel-Cox, *P* = 0.03). The adjusted OR for death (Table 2) by an early transfusion was 0.59 (95% CI, 0.29–1.20; *P* = 0.14).

**Figure 2.**
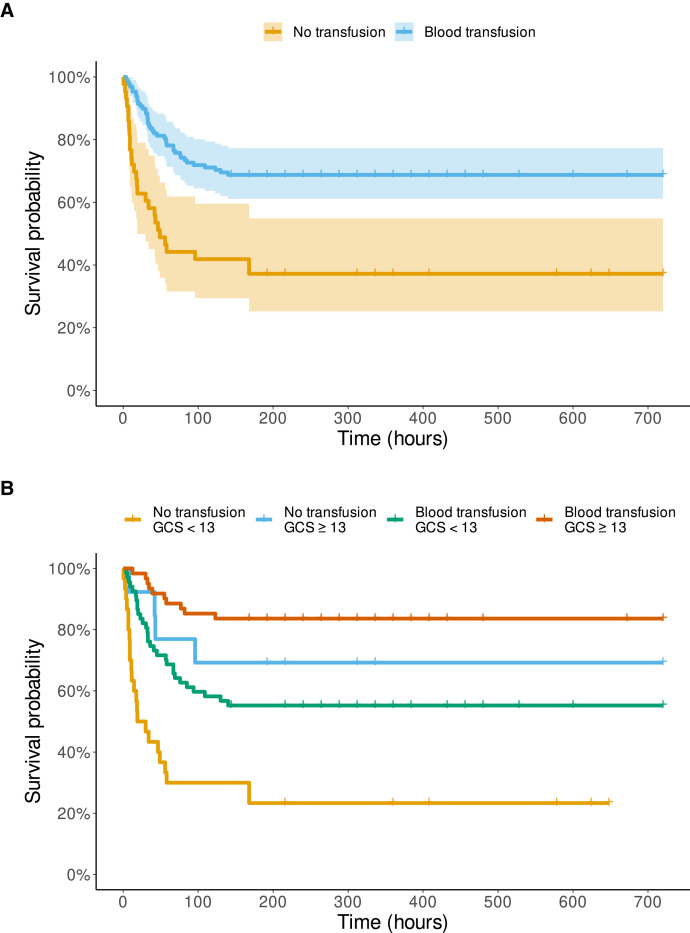
Time of survival in hours of the 171 patients with bacterial meningitis with admission hemoglobin < 6 g/dL with and without a blood transfusion any time during hospitalization vs. deaths within 30 days (= 720 hours) of admission. Glasgow Coma Scale (GCS) score on admission (3–15, best). Vertical lines on the curves indicate the exact time of follow-up for individual patients, if not 30 days. (**A**) Without stratification. The colored area represents 95% confidence intervals. (**B**) Stratified by severity of disease.

With regard to the effect of a transfusion at any time or no transfusion during hospitalization versus death within 30 days, the analysis gave very similar results (Figure 2) (log-rank Mantel-Cox, *P* < 0.0001). A transfusion reduced the OR for death (Table 2) to 0.27 (95% CI, 0.13–0.56; *P* = 0.0004) and, when adjusted for disease severity, to 0.30 (95% CI, 0.14–0.66; *P* = 0.002).

## DISCUSSION

Our results show an important benefit from early transfusion in reducing the risk of death and prolonging the time of survival of patients with BM and an admission hemoglobin level of < 6 g/dL. These are very positive results that point toward a concrete way to save lives almost already lost to BM. We think that, in our hospital, instead of < 5 g/dL, the cutoff for hemoglobin at < 6 g/dL could be a better guide to indicate the need of transfusion in patients with BM. The finding that transfusion lost its role as an independent predictor of death, when the Glasgow Coma Scale score < 13 points was included in the analysis emphasizes the fundamental prognostic importance of the severity at admission.^16^ This finding fits our earlier observation that if the patient arrives clinically “sufficiently” ill, with the cutoff lying somewhere around 13 points on the Glasgow Coma Scale, death is seldom avoided, regardless of the treatment administered.^16^

Aiming to understand the effects of transfusion on survival, we presumed that an early transfusion would be more important than a later one, and that its possible benefit would show within 6 to 7 days.^14^ Although aware of its less accurate nature, we also addressed a secondary question of whether a transfusion at any time during hospitalization would decrease 30-day mortality. This showed the same benefits of transfusion for reducing death and prolonging the time of survival—in fact, at even greater levels of significance (Figure 2, Table 2). Furthermore, transfusion remained an independent predictor of fewer deaths even when adjusted by disease severity on admission. This finding suggests that the mitigation of severe anemia in patients with BM is beneficial also after the first 2 days of treatment.

We have not found earlier data that would have explored specifically the role of transfusion in terms of vital prognosis of severely anemic children with BM. Instead, not giving blood to children with “serious febrile illness” (mostly malaria or sepsis) and severe anemia (using the limit < 5 g/dL) has been associated with more and earlier deaths—similar to our results.^1^ In adults with severe sepsis and septic shock, optimizing the hematocrit to 30 was one of the measures included in “early goal-directed therapy,” which decreased mortality significantly.^17^

We recognize limitations in our study. A secondary analysis of data cannot fully account for known and unknown confounding variables, in contrast to a randomized clinical trial.^18^ Of the potential known confounders, we tested the most prominent one, which is severity at admission (Glasgow Coma Scale score < 13 points).^4^ Including others, such as age younger than 1 year, treatment delay over 3 days, and patients who are severely underweight,^4^ did not change the results (data not shown). However, the reliability of the analyses with many independent variables is limited by the small number of the patients. A more precise analysis of the role of transfusion would have required information of the exact time the hemoglobin result was available, the precise indications and timing of transfusion in relation to death, and the reasons why it was not given. In our study, our questions left out the role of repeated transfusions. No data concerning potential harm of a transfusion were collected. When all data were not available from all patients, such as capillary refill time (Table 1), the results cannot be considered fully reliable. Acknowledging these handicaps, our data are a step forward in a difficult Third World setting in which many events materialize that are unimaginable in the Western World. The major benefit of our data is the large number of patients with the same confirmed diagnosis of BM. Other advantages include the exact recording of the time of death in relation to the institution of antimicrobial treatment. Undoubtedly, further and more precise studies on the questions addressed here (to our knowledge, for the first time) are warranted.

Early deaths of severely anemic children with BM who did not receive a transfusion emphasize the need for a rapid diagnosis of severe anemia, prompt relief by blood transfusion, permanent availability of this scarce resource, and a well-functioning blood bank. These needs are most relevant for areas with a high prevalence of severe anemia, such as sub-Saharan Africa.
